# Responsible Integration of Artificial Intelligence in Rapid Reviews: A Position Statement From the Cochrane Rapid Reviews Methods Group

**DOI:** 10.1002/cesm.70063

**Published:** 2025-11-24

**Authors:** Gerald Gartlehner, Barbara Nussbaumer‐Streit, Candyce Hamel, Chantelle Garritty, Ursula Griebler, Valerie Jean King, Declan Devane, Chris Kamel

**Affiliations:** ^1^ Cochrane Austria University for Continuing Education Krems Krems Austria; ^2^ Center for Advanced Public Health Methods RTI International Research Trianle Park NC USA; ^3^ Ottawa Hospital Research Institute Ottawa ON Canada; ^4^ School of Epidemiology and Public Health, Faculty of Medicine University of Ottawa Ottawa ON Canada; ^5^ Center for Evidence‐based Policy, School of Medicine Oregon Health and Science University (OHSU) Portland OR USA; ^6^ Evidence Synthesis Ireland & Cochrane Ireland University of Galway Galway Ireland; ^7^ School of Nursing and Midwifery University of Galway Galway Ireland; ^8^ Canada's Drug Agency Ottawa ON Canada

## 1 Background

Rapidly evolving artificial intelligence (AI) technologies are increasingly used to accelerate literature review processes. A recent review and evidence map identified almost 100 studies published since 2021assessing AI applications in evidence synthesis [1]. These technologies span from machine‐learning classifiers to generative large‐language models (LLMs). Recently, a preprint reported that a tool powered by LLMs autonomously reproduced and updated 12 Cochrane reviews in just 2 days [2], sparking debate about when and how AI can be used safely and effectively to support systematic and rapid reviews.

In this position statement, the Cochrane Rapid Reviews Methods Group outlines its stance on the use of AI in rapid reviews. Rapid reviews encompass various types of evidence synthesis, and while some AI tools have been developed for specific review types, such as qualitative evidence syntheses, most are designed for more general application across review methodologies.

The main recommendations are summarized in Textbox 1. They complement a recently released position statement by Cochrane and other evidence synthesis organizations on the use of AI in evidence synthesis [3].

Textbox 1 **Summary of Recommendations for AI Use in Cochrane Rapid Reviews.**

1

- Do not rely on AI to fully automate any step of a rapid review. Human oversight is essential to maintain methodological rigor and accountability.
- Use AI for quality assurance where it complements human judgment and helps mitigate risks associated with accelerated methods, such as single‐reviewer workflows by flagging missed studies, spotting inconsistencies, or detecting extraction errors.
- Be transparent about AI use and justify its use. Specify in the protocol which tools will be used, the tasks they will perform, and how their outputs will be verified. Clearly describe AI use in the Cochrane rapid review's “*AI Use Disclosure*” section. If using a generative large language model, document the model version and prompts applied.
- Demonstrate that the selected AI tool upholds the methodological rigor and integrity of the rapid review.
- Maintain author responsibility. AI is a support tool, but authors remain fully accountable for the accuracy, interpretation, and overall validity of the rapid review.
- Respect copyright and intellectual property. Ensure that AI tools are used in compliance with licensing terms.
- Adhere to RAISE principles and the Cochrane position statement on AI use in evidence synthesis to ensure that AI applications are ethical, transparent, and fit‐for‐purpose.

Abbreviations: AI = artificial intelligence, RAISE = Responsible AI use in Systematic Evidence Synthesis.

## 2 Semi‐Automation in Rapid Reviews

Semi‐automation of discrete steps in the evidence synthesis process —where algorithms assist but do not replace human reviewers—is not new. Cochrane, for instance, was an early adopter with the development of the randomized controlled trial (RCT) Classifier, a machine learning tool that identifies RCTs during abstract screening [4]. Semi‐automation plays a different role in rapid reviews than in traditional systematic reviews, where methodological certainty is typically prioritized. Because rapid reviews already balance rigor and timeliness, teams may be more willing to adopt efficiency‐enhancing tools sooner.

The advent of generative LLMs, such as ChatGPT [5] or Gemini [6], has substantially expanded the potential for AI to support tasks in evidence synthesis. Unlike earlier machine learning tools that required extensive task‐specific training data, LLMs can be deployed in zero‐shot settings—meaning they can be applied without prior training or fine‐tuning to a given task. This dramatically lowers the barrier to entry, offering a more accessible pathway for integrating AI into review workflows. Multiple studies have assessed the utility of generative LLMs to support the development of search strategies [7], literature screening [8, 9, 10], risk of bias assessment [11, 12], and data extraction [8, 13, 14, 15]. However, findings to date indicate highly variable performance ranging from high accuracy in some tasks to concerning errors in others [1]. In parallel, developers of literature review software have begun integrating LLMs into their products.

Importantly, in rapid reviews, AI has the potential not only to enhance efficiency but also to improve quality. Many rapid reviews rely on a single reviewer to perform key tasks—such as study selection, data extraction, risk of bias assessment, or certainty of evidence ratings, which heightens the risk of undetected errors. In these settings, AI can function as a scalable quality control tool, helping to identify inconsistencies, flag missing data, or suggest overlooked studies. For instance, Cochrane guidance for rapid reviews recommends switching to single‐reviewer screening if inter‐rater agreement during dual screening of abstracts is high [16, 17], which will likely miss some eligible studies [18]. In this context, AI may complement human judgment and mitigate the risks associated with single‐reviewer workflows. An AI‐integrated review software could re‐examine abstracts excluded during single‐reviewer screening, reducing the likelihood of erroneous exclusions. Another error‐prone step in the review process that could benefit from AI support is data extraction. Research shows that up to 50% of data elements extracted by humans include errors, depending on reviewer experience and topic complexity [19, 20]. The use of AI as a secondary reviewer for data extraction may improve data quality and reduce errors [21]. Ultimately, however, reviewers must decide whether the additional effort of using AI is feasible for their rapid review.

Despite its promising potential, AI also introduces risks. Specifically, generative LLMs can produce incorrect responses, fabricate data or references, perpetuate biases, and spread misinformation. To ensure that AI integration strengthens rather than undermines the credibility of rapid reviews, sustained human oversight, despite its own fallibility, must remain the core principle of any AI‐supported evidence synthesis effort. A recent review on the use of AI in evidence synthesis found that incorrect inclusion decisions of AI tools during screening ranged from 0% to 29% (median = 10%) and incorrect data extractions from 4% to 31% (median = 14%) [22].

## 3 Responsible Use of AI

The Responsible AI use in Systematic Evidence Synthesis (RAISE) guidance, developed through a multi‐stakeholder consensus process, provides foundational principles for the transparent, ethical, and scientifically sound integration of AI in evidence synthesis [23]. Cochrane has been actively involved in RAISE, both as a contributor and as an implementing organization, reflecting its commitment to ensuring methodological rigor in the face of rapid technological advancement. Members of the Cochrane Rapid Reviews Methods Group also participated in the development of RAISE, bringing expertise from the rapid review context where the pressure for speed makes AI adoption especially appealing—and potentially risky. RAISE emphasizes key principles such as transparency in reporting, human oversight, reproducibility, and fit‐for‐purpose evaluation. It cautions against overreliance on AI systems without robust validation and highlights the importance of disclosing when, how, and for which tasks automation has been used. As generative LLMs and other AI tools become increasingly accessible, adhering to RAISE principles will be essential to safeguard the trustworthiness and utility of AI‐assisted evidence synthesis.

When using AI tools, researchers also need to verify that any uploaded or processed material is permissible under fair use or equivalent scholarly exceptions and that the AI model itself complies with copyright and data protection standards. For instance, some professional or enterprise versions of generative LLMs explicitly guarantee that uploaded material will not be used for model training or redistribution, offering greater assurance of confidentiality and legal compliance. Transparent documentation of AI tool usage—including model version, purpose, and data inputs—should be maintained to uphold reproducibility, accountability, and ethical integrity in evidence synthesis.

## 4 What Does This Mean for Authors of Cochrane Rapid Reviews?

Cochrane, together with the Campbell Collaboration, JBI, and the Collaboration for Environmental Evidence, has endorsed RAISE in a position statement on the use of AI in evidence synthesis [3]. The statement emphasizes that evidence synthesists remain fully accountable for their work and must ensure ethical and legal compliance when using AI or automation. Any use of AI should be clearly justified, and the tool must be methodologically sound, ensuring that it does not compromise the trustworthiness or reliability of a review's findings. Importantly, all AI‐assisted tasks require human oversight, and any AI‐generated or AI‐informed judgments must be reported transparently in the final synthesis.

Authors should not use AI to fully automate an entire rapid review or any of its methodological steps. Doing so risks introducing errors, bias, and a lack of transparency, ultimately undermining the trustworthiness and reproducibility of the rapid review. Furthermore, such approaches violate established Cochrane methodological standards. Additionally, authors must continue to follow existing methodological guidance for Cochrane rapid reviews [16] and uphold Cochrane's standards of transparency, conflicts of interest, accountability, and scientific rigor [24].

The use of AI tools is acceptable—and even encouraged—when it serves to improve review quality. When resource constraints necessitate that a task, such as study selection or data extraction, is carried out by a single reviewer, an AI tool may be used to provide a secondary check or offer an independent suggestion. In this way, rapid review authors can introduce an additional layer of quality assurance with minimal extra effort. However, in all cases of AI use, a human reviewer must remain responsible for verifying all AI outputs and making final decisions.

Human reviewers must continue to resolve ambiguity, thoughtfully apply inclusion criteria, and interpret findings within the broader context of clinical relevance or policy implications. AI tools cannot be listed as authors, nor can they be accountable for their errors. Therefore, transparency regarding the use of AI is essential. The review protocol must document the intention to include AI during review production. The review methods and the new “*AI Use Disclosure*” section of Cochrane reports must clearly specify which tools were used, how they were applied, and what role they played in the review process. If reviewers used a generative LLM, the model version and prompts need to be documented. This includes a description of the extent of human oversight and any validation steps taken. It is critical that human reviewers retain all accountability, as AI systems lack the capacity for independent judgment and responsibility. While AI can support efficiency and quality assurance, human authors must be responsible for the final interpretation of evidence and all methodological judgments.

## 5 Future Possibilities

Fully autonomous evidence synthesis with AI remains a distant prospect. Retaining human oversight is not a shortcoming of AI, but a methodological imperative to safeguard the trustworthiness, transparency, and applicability of synthesized evidence.

Looking ahead, we are likely to see an increase in fully automated outputs—reviews that appear well‐written and methodologically sound on the surface but lack the depth of critical evaluation required for high‐stakes decision‐making. Without proper human oversight, this could unleash a flood of low‐quality reviews that are difficult to detect. Vigilance by peer reviewers and editors will be paramount. While increased automation in rapid reviews is likely inevitable and welcome, the transition must be carefully managed with strict adherence to rigorous conduct and reporting standards. To ensure that rapid reviews serve the needs of both science and society, active participation of human expertise will remain indispensable for the foreseeable future.

Research in this area is advancing rapidly, and the views expressed here reflect the position of the Cochrane Rapid Reviews Methods Group at the time of writing. As the evidence base evolves, this statement will be reviewed at least biannually and updated as needed. We strongly encourage rapid review authors to acquaint themselves with the most recent versions of RAISE [23] and the Cochrane position statement on AI use in evidence synthesis [3].

## Author Contributions

**Gerald Gartlehner:** conceptualization, writing – review and editing, writing – original draft, project administration. Barbara **Nussbaumer‐Streit:** conceptualization, writing – review and editing. Candyce **Hamel:** conceptualization, writing – review and editing. Chantelle **Garritty:** writing – review and editing, conceptualization. Ursula **Griebler:** writing – review and editing, conceptualization. Valerie Jean **King:** conceptualization, writing – review and editing. Declan **Devane:** conceptualization, writing – review and editing. Chris **Kamel:** conceptualization, writing – review and editing.

## Conflicts of Interest

All authors are co‐convenors of the Cochrane Rapid Reviews Methods Group; G.G. and H.C. are co‐convenors of the Cochrane Artificial Intelligence Methods Group.

## Data Availability

Data sharing is not applicable to this article, as no new data were created or analyzed in this study.
